# Epstein-Barr virus from Burkitt Lymphoma biopsies from Africa and South America share novel LMP-1 promoter and gene variations

**DOI:** 10.1038/srep16706

**Published:** 2015-11-23

**Authors:** Haiyan Lei, Tianwei Li, Bingjie Li, Shien Tsai, Robert J. Biggar, Francis Nkrumah, Janet Neequaye, Marina Gutierrez, Sidnei Epelman, Sam M. Mbulaiteye, Kishor Bhatia, Shyh-Ching Lo

**Affiliations:** 1 Center for Biologics Evaluation and Research, Food and Drug Administration, Silver Spring, Maryland; 2 Bethesda Maryland, formerly NCI; 3 Noguchi Memorial Institute, Accra, Ghana; 4 Department of Child Health, University of Ghana, Accra, Ghana; 5 Laboratorio Stamboulian, Buenos Aires, Argentina; 6 Department of Pediatric Oncology, St Marcelina Hospital, Sao Paolo, Brazil; 7 Division of Cancer Epidemiology and Genetics, National Cancer Institute, National Institutes of Health, Rockville, Maryland

## Abstract

Epstein Barr virus (EBV) sequence variation is thought to contribute to Burkitt lymphoma (BL), but lack of data from primary BL tumors hampers efforts to test this hypothesis. We directly sequenced EBV from 12 BL biopsies from Ghana, Brazil, and Argentina, aligned the obtained reads to the wild-type (WT) EBV reference sequence, and compared them with 100 published EBV genomes from normal and diseased people from around the world. The 12 BL EBVs were Type 1. Eleven clustered close to each other and to EBV from Raji BL cell line, but away from 12 EBVs reported from other BL-derived cell lines and away from EBV from NPC and healthy people from Asia. We discovered 23 shared novel nucleotide-base changes in the latent membrane protein (LMP)-1 promoter and gene (associated with 9 novel amino acid changes in the LMP-1 protein) of the 11 BL EBVs. Alignment of this region for the 112 EBV genomes revealed four distinct patterns, tentatively termed patterns A to D. The distribution of BL EBVs was 48%, 8%, 24% and 20% for patterns A to D, respectively; the NPC EBV’s were Pattern B, and EBV-WT was pattern D. Further work is needed to investigate the association between EBV LMP-1 patterns with BL.

Epstein-Barr virus (EBV), considered the first human tumor virus, was first discovered in Burkitt lymphoma (BL) tumor cells in 1964^1^. It was subsequently linked to other lymphoid cancers (Hodgkin lymphoma^2^ and nasal T cell lymphomas^3^) and to epithelial cancers (nasopharyngeal carcinoma (NPC)^4^,^5^ and gastric cancer^6^,^7^), and it was declared a class 1 carcinogen in 1997^5^. EBV was shown to mostly be asymptomatic, particularly in developing countries^8^, and to circulate as lifelong infection in up to 95% of the world’s adult population^9^, being rarely detected in cancer. In contrast to its ubiquitous nature^10^, the cancers linked to it often have a regional incidence distribution^11^. For example, BL occurs mainly in African children living in equatorial regions^12^, while NPC occurs most commonly in Asian and North African adults. This regional distribution, coupled with differences in ages at clinical presentation for different cancers, suggested that there might be different high-risk EBV genetic variants influencing the observed epidemiological and clinical EBV-associated tumor patterns^13^,^14^. If so, the discovery of high-risk EBV variants might direct public health or clinical strategies to prevent EBV-associated malignancy^15^,^16^,^17^.

However, no simple correlation between EBV genetic variation and EBV-associated cancers has been presented^18^,^19^,^20^,^21^,^22^,^23^,^24^,^25^,^26^,^27^,^28^, although EBV is known to exist as two genetic types (Type 1 or 2)^29^, which both immortalize cells and harbor significant genetic variability in EBV latent genes^29^. Technical constraints have limited studies to examining genetic variation in short sequence stretches in single EBV genes rather than to study of entire or multiple genes or the whole EBV genome^18^, while lack of primary BL samples from different geographical areas has also limited ability to study tumors from different areas. Recent technological advancements have enabled whole EBV genome sequencing, first successfully done in 1984 with wild-type (WT) obtained from an immortalized cell line infected by EBV from a patient with infectious mononucleosis, B95-8 (V01555.2)^30^ and subsequently expanded to include EBV from three BL cell lines (AG876^31^, Akata and Mutu^32^), EBV from NPC tumors^33^,^34^,^35^,^36^ and BL tumor-derived cell lines^36^. The resulting EBV genomic library is extensive and provides the potential to discover high-risk carcinogenic variants^35^,^37^,^38^, but currently does not include data from primary BL tumor samples and may be biased towards viruses that are well adapted to grow *in vitro*.

In the present study, we directly sequenced DNA from 12 primary BL biopsy samples from Ghana, Brazil and Argentina using Illumina-MiSeq platform^37^, and aligned the reads obtained from each BL sample to the WT EBV (NC_007605) reference sequence. Our objective was to find novel putative BL-associated EBV genetic traits. We compared sequences from the 12 BL EBVs with the publically available, well-annotated EBV sequence data from benign and malignant tumors, mainly using data registered with National Center for Biotechnology Information (NCBI), to identify genetic commonalities and to gain insights about new ways to categorize EBV variants.

## Results

### EBV sequences from primary Burkitt lymphoma biopsies were Type 1

Our results increase the number of whole EBV genomes from BL from 13 to 25 in NCBI, and are the first EBV sequence results from primary BL tumors. They complement the results from tumor-derived BL cell lines, which might be biased by over-selection of viruses that are better adapted to grow *in vitro*. Detailed sequencing results are available in supplemental results and supplementary Tables 1 and 2. The median EBV genome size found in the EBV from BL samples was 170,597 bp (range: 163,639 to 171,595 bp) and the average coverage of the genome in each sample was 30 times (range 15 to 70). Consistent with prior results, we found a high median EBV copy number per BL tumor cell (median: 50; range 28–95). Similarly, viral sequences were clonal, showing unremarkable intra-tumor heterogeneity at only one possible position in three of the 12 tumors examined (Supplementary Table 3).

The BL EBVs in our series were all Type 1. By comparison, nine of the previously published EBV genomes from tumor-derived BL cell lines (Asia (2), Kenya (4), Nigeria (1), North Africa (1) and Africa unspecified (1) were associated with Type 1 and four were associated with Type 2 EBV (from Nigeria, Kenya, Papua New Guinea, and Ghana). Our combined results suggest that 84% of BL is associated with Type 1 EBV and 16% with Type 2 EBV. All 16 NPC EBVs (from China or Hong Kong) were associated with Type 1.

### Phylogenetic analysis of 12 BL EBV genomes versus 100 EBV genomes shows distinct patterns

**Figure 1:**
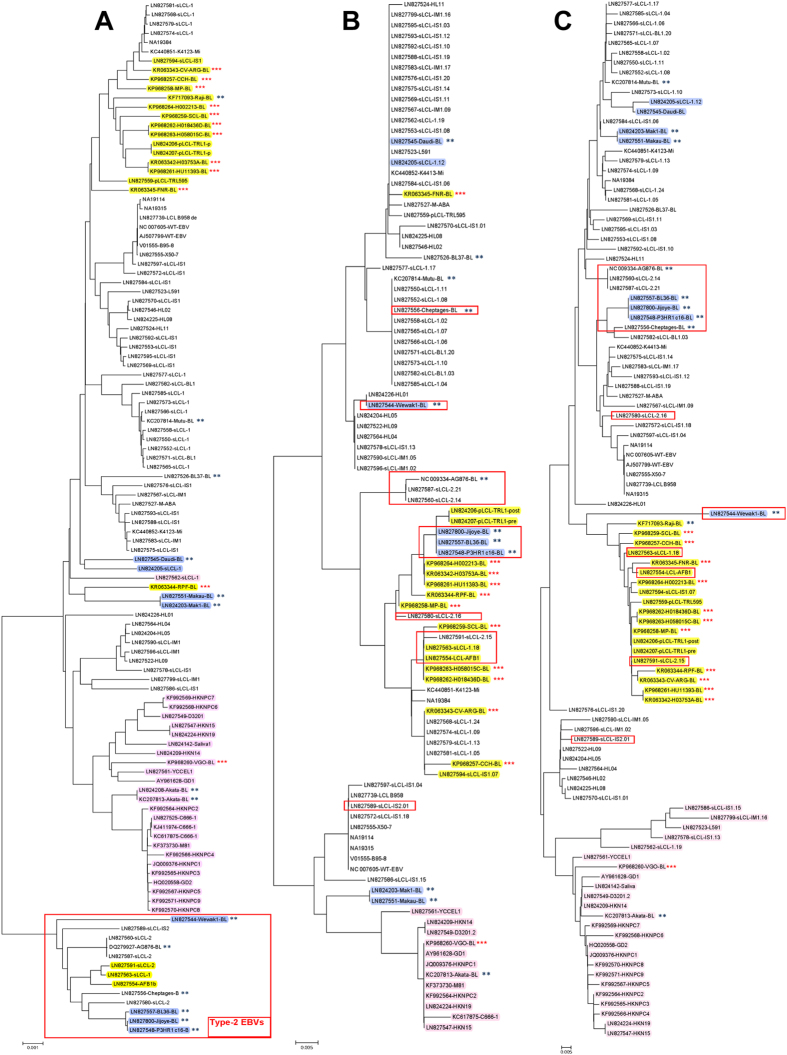
Phylogenetic analysis based on heterogeneity of EBV genome sequences (A), amino acid sequences of EBNA-1 (B) and amino acid sequences of LMP-1 (C). The 12 BL-EBVs genomes sequenced from BL tumors in our study are marked with three red stars. The 13 BL-EBV genomes previously sequenced from BL cell lines are marked with two blue stars. Type 2 EBVs are boxed in red rectangle(s). EBV genomes are classified into Pattern A (yellow), Pattern B (pink), Pattern C (blue) and Pattern D (no color) for the variations/mutations identified in the promoter region and the N-terminus of LMP-1.

Full-length phylogenetic analysis of our 12 BL EBVs and the 100 public EBVs genomes showed that 10 of 12 BL EBVs clustered together, while two BL EBVs, both from Brazil (KP 968260-VGO and KR63344-RPF), arrayed far from the first 10 (Fig. 1a). The 10 similar BL EBVs were close to WT-EBV and to EBV sequenced from healthy individuals in the United States and Kenya (e.g., K4123Mi and NA19384)^37^,^38^. Of the two different, Brazil BL EBVs, one (KP968260-VGO) was close to EBVs from Asia, including from 16 NPC from China and Hong Kong, from Akata BL tumor-derived cell line, obtained from a Japanese patient, and from healthy people in Asia. The ethnicity of this Brazil subject (KP968260-VGO) was not recorded. The EBV from this Brazil subject and the EBV genomes reported from Asia arrayed distinctly from the 11 BL EBVs from biopsies (Fig. 1a). The second different BL EBV (KR063344-RPF) clustered away from the EBVs reported from Asia, but it was closer to three EBVs from tumor-derived BL cell lines registered in the NCBI (LN827551-Makau, LN824203-Mak1, and LN827545-Daudi (Fig. 1a), as well as one EBV from a healthy individual from Kenya (LN827562).

### Phylogenetic analysis of imputed amino acid sequences reveals most variation in EBNA-1 and LMP-1 proteins

Phylogenetic analysis of amino acid sequences imputed for EBV nuclear antigen 1 (EBNA-1) (Fig. 1b) and LMP-1 (Fig. 1c) showed similar phylogenetic clustering as we found using full-length whole EBV genomes, albeit with minor variations. The 10 similar BL EBVs were also close to each other on both EBNA-1 and LMP-1 imputed protein sequences. Within this group, however, two distinct sub-clusters were also detected using EBNA-1 (Fig. 1b), that were not observed with LMP-1 amino acid sequences (Fig. 1c). EBNA-1 and LMP-1 protein sequences from the two different, Brazil BL EBVs (KP968260-VGO and KR063344-RPF) showed phylogenic separation as described in the full-length EBV genome analysis but in different ways. The KP968260-VGO EBV was closer to the Asian NPC and non-NPC EBVs in both EBNA-1 and LMP-1 comparisons, while the KR063344-RPF EBV was closer to the similar BL EBVs, while KR063345-FNR was the outlier in EBNA-1 comparisons, clustering closer to LN827545-Daudi (Fig. 1b) but not in the LMP-1 comparisons (Fig. 1c).

Thirteen BL EBVs, all sequenced from BL tumor-derived cell lines, have been previously reported (Akata, Mutu, Raji, AG876, jijoye, Wewak1, P3HR1, c16, Daudi, Cheptages, BL36, BL37, and Makau). One of these BL EBVs (Raji) aligned most closely to our 11 similar BL EBVs for both full-length EBV genomes and LMP-1 protein sequences (Fig. 1a,b). Comparisons for EBNA-1 sequence of Raji was not included (Fig. 1b) because it is not annotated in the NCBI database. Three BL EBVs (jijoye, P3HR1, BL36 and Wewak1), all Type 2 EBV, were different from our BL EBVs, in the EBNA-1 and LMP-1 phylogenetic relationships. The remaining 8 BL EBVs from BL tumor-derived cell lines aligned separately, forming potentially three sub-clusters. The Brazil outlier EBV of unknown ethnicity (KP968260-VGO) arrayed close to the Asian Akata EBV in the EBNA-1 and LMP-1 analyses as well (Fig. 1b,c).

### Analysis of nucleotide sequences reveals common sequence variations in the 11 of 12 BL EBV genomes

**Figure 2:**
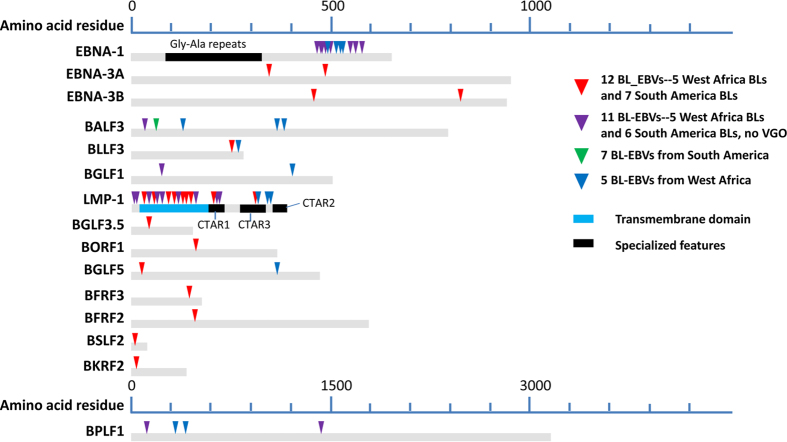
Shared amino acid (aa) changes identified in EBVs from BL tumors. Positions of common aa changes due to non-synonymous SNVs identified in the 15 EBV genes with known or putative functions are marked by color arrows. The positions of common aa changes shared by all 12 BL-EBVs studied are marked with red arrows, those shared by 11 of the 12 BL-EBVs, except VGO–EBV, are marked with purple arrows. Most of these aa changes (red arrows) are also observed in NPC-EBVs. Green arrow indicates the position with common aa change shared by 7 South America BL-EBVs. Blue arrows indicate the positions with common aa changes shared by 5 West Africa BL-EBVs. Transmembrane domain of LMP-1 protein is illustrated using light blue. Black bars mark the regions with specialized features of EBNA-1, and LMP-1.

Whole genome sequence alignments revealed extensive nucleotide variation in all of the 12 BL biopsy EBV genomes compared to the WT-EBV reference (Supplemental Figure 2a). The density of variations per genome region was higher when the genomes were compared to Type 1 EBV GD1 associated with NPC (Supplemental Figure 2b), and substantially higher when compared with Type 2 EBV AG876 (Supplemental Figure 2c). Compared to the WT-EBV, the 12 BL biopsy EBVs shared overall 67 common nucleotide variations, but this number increased to 94-shared variations, when the outlier KP968260-VGO EBV was excluded. Analysis of the 95 coding DNA sequence (CDS) in EBV genome revealed 36 shared common non-synonymous amino acid variations occurred in 15 EBV genes (Fig. 2). Some of the variations were consistent with a geographical association rather than a BL-association. For example, a variation in BALF3 was found only in the 7 South America BL-EBVs, while several variations were found in different genes only in the 5 West Africa BL-EBVs (Fig. 2).

The analysis of imputed common amino acid changes shared by BL EBVs in all CDS of the EBV genome revealed hypervariable regions mostly in EBNA-1 and LMP-1 (Fig. 2). However, since most of the shared amino acid changes in EBNA-1 in the BL EBVs were also found in EBV from non-diseased individuals, while those in LMP-1, particularly in the N-terminus, appeared to be novel and unique to BL EBVs we focused our detailed comparisons on the LMP-1 promoter and its N-terminal region of the gene.

### Sequence analysis of 12 BL EBVs reveals novel changes in the LMP-1 promoter and coding region

**Figure 3:**
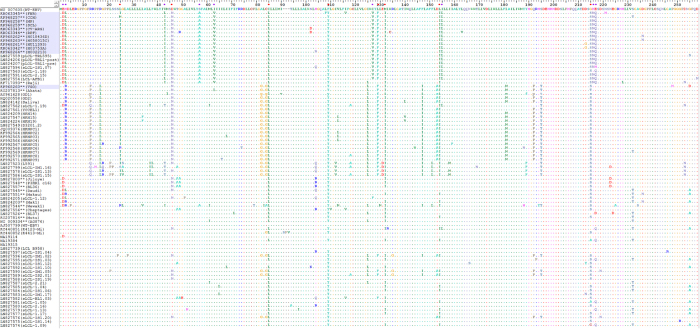
Amino acid (aa) sequence alignment (1-235 aa) for LMP-1 proteins (387 aa) deduced from genomes of BL-EBVs and EBVs available in NCBI database. There are overall 22 common aa changes observed in LMP-1 protein from BL-EBVs. Positions of the 9 out of the 22 common aa changes uniquely shared by the similar 11 BL-EBVs studied, but not VGO, are marked with purple dots (E2D, H3L, S57A, I63V, I124V, I152L, H213N and E214Q). Positions of the 8 aa out of the 13 common aa changes in the N-terminus are marked as red dots in this figure: L25I, D46N, I85L, F106Y, L126F, M129I, L151I and G212S. These changes were also present in essentially all NPC-EBVs. The other 5 common aa changes observed in all 12 BL-EBVs and essentially all NPC-EBVs are not shown in the Figure: S309N, Q322N, Q334R, L338S and S366T.

Analysis of the 2.1 kb sequence stretch covering LMP-1 promoter and N-terminus of coding sequence revealed a total of 51 common nucleotide variations in our 12 new BL EBVs: 19 were in the promoter region and 32 in the coding region as compared with WT-EBV. Importantly, 23 common nucleotide variations (12 in the promoter region and 11 in the coding region were novel) were shared by the 11 similar BL-EBVs (Fig. 3) and not by the outlier KP968260-VGO or any of the NPC-EBVs or non-NPC EBVs from Asia (Fig. 3). We separately confirmed these novel sequences in our 11 BL biopsy EBVs using Sanger sequencing of PCR products amplified from the target region. Eleven of the 23-nucleotide changes led to amino acid changes, 10 of which coded 9 novel amino acid changes in the N-terminal region of LMP-1 and one nucleotide change located in the second intron (Fig. 3). The 9 novel N-terminus amino acid changes were not seen in the 12 BL EBV genomes from the BL tumor-derived cell lines, however, 7 out of the 9 amino acid variations imputed [Table t1]were found in the EBV sequenced from the Raji cell line^36^ (Fig. 3 and Table 2).

**Figure 4:**
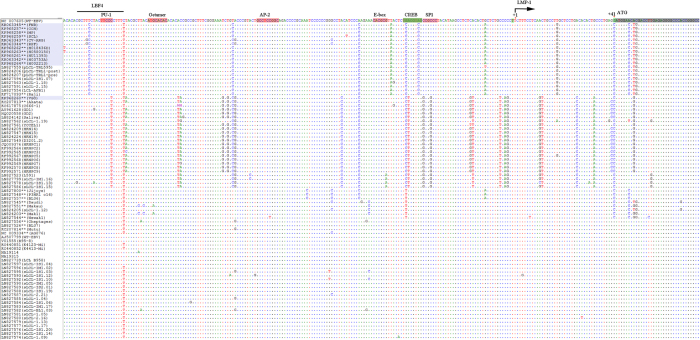
Alignment of nt sequences in LMP-1 promoter region in ~220 nt upstream of its coding region. The alignment is corresponding to 169239–168992 positions in the reference genome of WT-EBV including part of the LMP-1 coding region (25 nt shadowed with black) with marked ATG initiation codon (position 169017). The transcription starting site is marked as +1 (position 169058). The sequences recognized for functions associated with transcription factors (ref. 27) are marked with pink or green shadow. The 5 common variations or mutations shared by 11 of the12 BL-EBVs, but not by VGO or any NPC-EBVs are T-172C, T-50A, A-39C (in CREB), G-12A and T+18G. In this particular region, there are additional 5 common variations or mutations, C-158T, T-114C, A-63C, G-44C and G+41C, shared by all 12 BL-EBVs and essentially all NPC-EBVs.

**Figure 5:**
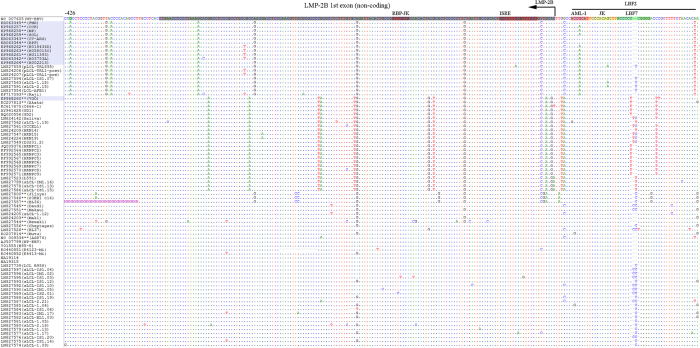
Alignment of nt sequences in LMP-1 promoter region -183 to -428 upstream of the transcribing region. The alignment is corresponding to 169485 to169240 positions at the very end of WT-EBV reference genome, neighboring the terminal repeats (TR). The sequences recognized for functions associated with transcription factors (ref. 27)) are marked with pink or green shadow. The region shadowed with black (from 169294–169448 in the WT-EBV) was identified as LMP-2B 1^st^ exon (noncoding) (ref. 23 in the manuscript). The 7 common variations/mutations (G-426A, T-412G, C-410A, G-376A, A-354G, and A-184T) were shared by the 11 similar BL-EBVs, but not by VGO and any NPC-EBVs. One additional common mutation (G-227A) located in AML1 of LBF2 was shared by 9 BL-EBVs is, not present in VGO and any NPC-EBVs. There are 3 additional common variations or mutations, A-314G, C-272G and T-242C, shared by all 12 BL-EBVs and essentially all NPC-EBVs.

**Figure 6:**
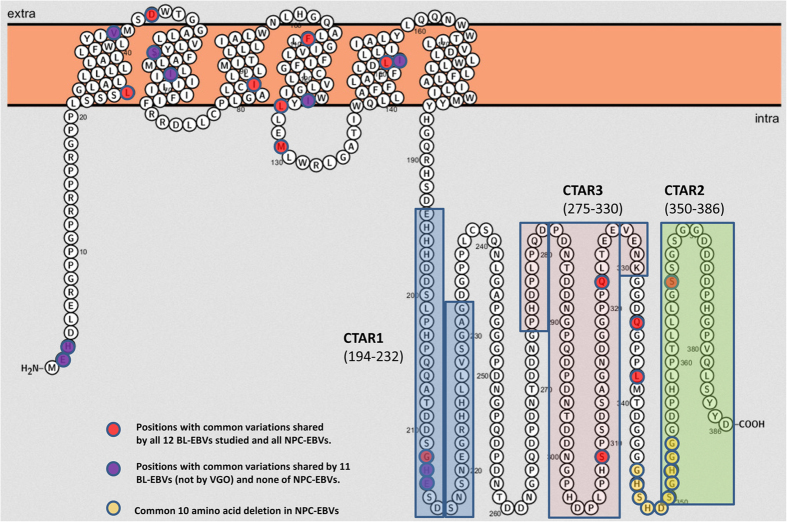
Graphic depiction of aa changes in LMP-1 protein. The positions of 9 common aa changes (resulted from10 nt changes in the coding region) uniquely shared by 11 of 12 BL-EBVs, except VGO, are marked purple. None of these 9 aa common changes are observed in NPC-EBVs. They are located in cytoplasmic domain (2 aa), intramembrane domain (5 aa) and CTAR1 (2 aa) of LMP-1 protein. The positions of 13 common aa changes shared by essentially all 12 BL-EBVs studied as well as NPC-EBVs in NCBI database are marked red. Positions of 10 common aa deletions found in NPC-EBVs are marked yellow. C-terminal activation regions 1–3 (CTAR1-3) of the protein are marked by rectangle.

The 12 novel nucleotide variations in the LMP-1 promoter were: G-426A, T-412G, C-410A, G-376A, A-354G, G-227A, A-184T, T-172C, T-50A, A-39C, G-12A, and T+18G. All 12 nucleotide variations imputed in the LMP-1 promoter were found in the EBV from Raji cell line^36^. Four of the 12 variations were located in LMP-1 regulatory elements AML1 (G-227A), LBF2 (A-184T), LBF4 (T-172C), and CREB (A-39C) (Figs 4 and 5, Table 2) hint at a possible role in altering LMP-1 promoter function. The 9 N-terminal amino acid changes were located in the cytoplasmic domain (2 amino acids), intramembrane domain (5 amino acids) and C-terminal activation regions (CTAR) 1 (2 amino acids) (Fig. 6), but their functional significance was not evaluated in our study, and it is unknown.

### Alignment of 112 EBV genomes in the LMP-1 promoter region reveals four novel clustering patterns

When we aligned the LMP-1 promoter and coding region for the 12 BL EBVs and other 100 published EBV genomes registered in NCBI (Supplementary Table 4), including 75 genomes recently reported by Palser *et al.*^36^, four strikingly distinct patterns of nucleotide variations, designated A to D, in this region were observed (Figs 3, 4, 5). Pattern A: Characterized by the 23 novel nucleotide sequences in the promoter region and the coding region of LMP-1 that were shared by the 11 similar BL-biopsy EBVs as noted above. An identical or highly similar pattern of common 12 nucleotide variations in the promoter region and 9 amino acid changes in the N-terminus of LMP-1 gene were found in Raji-EBV and 7 EBVs from other lymphoid conditions that have been recently published^36^ (Table 2). Four of the 7 non-BL EBVs were from patients with post-transplant lymphoproliferative disease (PTLD) in the US and Australia. The other 3 were from type 2 EBVs. This pattern was not observed in the outlier Brazil BL (KP968260-VGO) EBV, or in any EBV from NPC or in healthy individuals from Asia. Overall, Pattern A was observed in 19/112 (17%) EBV genomes reported to NCBI, but found in about half of BL cases (12 of 25, 48%, including all but one of our new cases). In contrast, it was less common in established lymphoblastoid cell line (LCL) (1 out of 4, 25%), PTLD EBVs from USA and Australia (4 of 19: 21%) and spontaneous lymphoblastoid cell line (sLCL) EBVs from Kenya (2 of 30: 6%). Notably, three Pattern A EBVs were Type 2 EBVs (two Kenyan sLCL and one LCL of unknown origin).

Pattern B: Characterized by 13 common nucleotide variations at positions G-372A, C-356A, C-329T, G-328A, C-315T, A-286G, G-284T, G-240A, A-238G, G-234T, G-233A, C-207T and C-199T. Pattern B was observed in 28/112 (25%) EBVs, but it also appears to be an Asian type EBV, as it was shared by all NPC-EBVs from China and Hong Kong Asia, including 2 of 25 (8%) BL EBVs, Akata (Japan) and KP968260-VGO (Brazil), which clustered with the NPC EBVs in phylogenetic analyses, as well as with EBV from saliva of a healthy person presumed to be Asian^36^, and 5 sLCL from Asia. Pattern C: Characterized by novel E2D amino acid change in LMP-1 coding region plus G-44T and G+41C nucleotide changes in the LMP-1 promoter and other isolated variations. The Pattern C shared E2D amino acid change and G+41C nucleotide change with pattern A, but lacked the other characteristic Pattern A mutations/variations. In addition, the Pattern C had a unique common variation at position G-44T within the regulatory CRE element of LMP-1 promoter. Pattern C was present in 8 of 112 (7%) EBVs, including from 7 BL tumor-derived cell lines (P3HR1, jijoye, Daudi, Makau, Mak1, BL36 and Wewaki) and one sLCL from Kenya. Pattern C was not present in EBV from NPC or healthy people from Asia. Pattern D: Was similar to the reference WT EBV. It was observed in 58 of 112 (52%) in the analyzed EBV genomes. The majority of Pattern D EBVs were from sLCLs (30 of 58, 52%), but it also occurred in diverse lymphoid conditions: 13 of 58 (22%) from PTLD, 7 of 58 (12%) from Hodgkin lymphoma, 6 of 58 (10%) from BL. Pattern D included both Type 1 and 2 EBVs.

## Discussion

Our study doubles the number of published EBV genomes from BL to 25, and presents the first set of results obtained by directly sequencing DNA from primary BL biopsies. The study of primary tumors fills the main gap in the picture of EBV diversity found in BL, which has hitherto relied on tumor-derived BL cell lines and carried the risk of over-selecting for viruses that are well adapted to grow *in vitro*. We showed that BL EBVs from Ghana and South America, with the exception of one, phylogenetically clustered together, near WT-EBV and EBV sequenced from healthy and diseased individuals in the United States and Africa, but distant from EBV from NPC and healthy people reported from Asia. We discovered 23 novel nucleotide base substitution signature in the LMP-1 promoter and coding region (associated with 9 amino acid changes in the LMP-1 protein) that was shared by 11 of 12 similar BL EBVs from Ghana and South America. Importantly, highly similar or identical changes also occurred in one EBV sequenced from a tumor derived BL cell line (Raji) from Nigeria, in four Australian/American PTLDs and three LCLs of type 2 EBVs, including two from Kenya. These results suggest that the novel signature is not unique to BL, but it is most prevalent in BL EBVs, occurring in 48% of BL EBVs compared to 7% of 87 other analyzed EBVs genomes. If validated in large, well-selected series, this signature may prove useful as an EBV genetic marker for BL.

Our detailed analysis of the LMP-1 promoter and coding sequences for 112 EBV genomes in NCBI revealed four striking patterns of nucleotide substitutions in the analyzed EBV genomes, tentatively designated Patterns A to D. These patterns were independent of variations in EBNA2 that are used to classify EBV into subtypes^29^ and different from the similarly designated patterns proposed by Sandvej *et al.*^27^, based on LMP-1 Xho I polymorphism and the 30-bp deletion and a limited but different set off LMP-1 promoter base substitutions. While Sandvej’s patterns do not appear to be particularly useful as genetic markers of EBV variants associated with specific EBV-related malignancies^27^, our finding that of genetic patterns with a variable distribution in some EBV-associated malignancies is intriguing. Notably, the pattern in the 25 BL cases was 48%, 8%, 24% and 20% for Pattern A through D, respectively. Of the 19 EBVs with Pattern A LMP-1, 12 (63%) were from BL samples from different continents, while the other 7 Pattern A EBVs included 4 PTLDs (US/ Australia) and 3 Type 2 sLCL/LCL (2 from Kenya; one of unknown) from different geographical areas. In comparison, Pattern B was found almost only in Asia and in both healthy and disease samples. In this context, it is important to note that there were 18 EBV genomes marked as NPC EBVs (AB850643 to AB850660) in NCBI database that were excluded in our full-length EBV genome analysis because they lacked references of publication, lacked detailed annotation and description of origin showed clearly Pattern B in the LMP-1 analysis. Whether Pattern A is associated with BL and Pattern B with NPC cannot be determined from our analysis, but disease associations will become clearer when case-control studies with representative controls are done.

Our finding of a novel signature in the LMP-1 promoter and gene was unexpected. The sequence variations in LMP1 gene promoter and/or coding sequences may play a role in the immune regulation, affect LMP1 signaling through interacting proteins in BL tumors or they may act as a strain marker. LMP-1 is a viral oncogene and it is expressed in some EBV-associated malignancies, such as Hodgkin lymphoma and NPC, but not in BL^39^. Thus, our finding might be a clue about an important role LMP-1 plays in BL carcinogenicity as well. There is some evidence that mutations in LMP-1 regulatory sites could reduce the responsiveness of the LMP-1 promoter to transcription factors, which might favor survival and promotion of carcinogenesis by mutated variants^40^. For example, Jansson *et al.’*s report^41^ that a single base substitution (G-44T) within the CRE element of the LMP-1 promoter of EBV from the P3HR1 cell line, an African origin tumor-derived BL cell line, altered factor-binding properties of LMP-1 promoter sequence (LRS) and reduced activation of the LMP-1 promoter as compared the corresponding B95-8 sites provides support for this reasoning. Our finding of 2 nucleotide variations(A-39C and G-44C, the latter is also observed in essentially all NPC-EBVs), albeit different, located within and potentially disrupting the LRS CRE of the LMP-1 promoter (Fig. 3B) in the 11 similar BL EBVs is consistent with the hypothesis that substitutions in regulatory sites may be a feature of carcinogenic EBV variants. However, our analysis of flanking regions that may be controlled by the LMP-1 promoter, such as LMP-2A, is incomplete, hence alternative functional explanations are possible. The LMP-2A gene, which is expressed by episomal viral genome, such as is found in BL^42^, modulates lytic viral activation *in vitro*, and non-expression has been correlated with reduced transforming ability of EBV^42^.

The LMP-1 pattern A has apparently existed before the evolutionary diversion of Type 1 and Type 2 EBV, based on its presence in EBV Type 1 or Type 2. Since this genetic pattern has apparently been preserved in individuals living in many different geographical areas in such a long period, it may likely be functionally important. However, its role in BL carcinogenicity could be indirect because Pattern A was not seen in Type 2- associated BL EBVs, despite its high frequency in Type I BL EBVs.

Strengths of our approach include the use of primary BL tumor samples from different geographical areas to sequence whole EBV genomes. which improves and complements previous efforts that were limited to studying variation in short stretch sequences of single EBV genes from tumor-derived BL cell lines^11^,^18^,^43^. The use of primary tumor samples reduces risk of bias towards viruses adapted to grow *in vitro* when tumor-derived BL cell lines are used. The main limitation of the study is lack of representative control samples to more critically evaluate disease-specific associations. Instead, we used whole EBV genome sequence data from the NCBI, which includes healthy and diseased populations from all continents, although not from exactly the same areas.

To summarize, we present the first set of EBV genomes sequenced from primary BL samples from different geographical areas. We showed that BL EBVs were closer to each other and distant from NPC EBVs, and we discovered novel LMP-1 promoter and gene changes that may prove useful for classifying EBVs into four different groups. Our findings justify case-control studies to validate the novel LMP-1 variants and measure disease-specific associations with BL and other EBV-associated cancers.

**Note:** During the review of the paper, we sequenced 2 additional BL biopsies (VA and SG) obtained from Argentina in South America, thereby increasing the number of WBV whole genomes in NCBI to 27. Both EBV genomes showed Pattern A in LMP-1 analysis with the characteristic 23 nucleotide changes in its promoter and the coding gene, thus Pattern A EBV genotype was observed in 13 out of 14 EBVs sequenced directly from BL tumors. The full-length sequences of these 2 EBV genomes have been submitted to NCBI (accession numbers: VA KT001102; SG KT001103).

## Methods

### Study population

The BL samples were fresh-frozen biopsies obtained mostly from the abdomen of children with BL aged less than 15 years in Ghana (N = 5)^44^,^45^, Brazil (N = 6) and Argentina (N = 1)^46^ (Table 1) enrolled in historical studies performed by investigators at the National Cancer Institute. All diagnoses of tumor biopsies were confirmed histologically.

## Ethics Review

The current study was carried out in accordance with the approved guidelines. The historical studies were conducted after ethical approval from the local institutions (Korle Bu University, the Hospital AC Camargo, Sao Paulo, Brazil, and CIIH Domingos Boldrini, Campinas,Brazil, and Hospital Nacional de Pediatria “Juan Garrahan,” both in Buenos fires, Argentina), and subjects gave informed consent to participate. The current study received exemption from the Office of Human Subjects Research at the National Institutes of Health to use de-identified samples. Sequencing study of previously frozen DNA samples from BL biopsies was conducted under FDA Research Involving Human Subjects Committee (RIHSC) protocol #10-008B entitled "Detection of Infectious Agents in Previously Frozen Blood Samples from Patients with Various Illnesses and Healthy Blood Donors".

### Sample preparation and EBV genome sequencing

DNA was extracted from tumor samples for molecular studies as previously described^46^. DNA was directly sequenced by Illumina-MiSeq as previously described^37^. Briefly, approximately 50 ng of DNA extracted from each of the BL tumor samples was subjected to DNA library construction using the Nextera DNA Sample Prep Kit (Illumina) through tagmentation and 5-cycle polymerase chain reaction amplification according to the manufacture’s protocol. The average DNA library has insert sizes ranging from 250 to 1000 base pairs (bp) with the peak around 500 bp. Sequencing was conducted using Illumina MiSeq Reagent Kit V2 (500 cycles for the 2 × 250 bp pair-end sequencing) and the raw reads were processed following the previously described workflow (Supplementary Figure 1)^37^.

### EBV genome assembly, alignment, and phylogenetic analysis

The sequences from each of the 12 samples were filtered at a Q30 phred score and trimmed to remove low quality base reads (with read error probability score >0.05, <2 ambiguities in the reads or read-lengths of less than 15 bp) using CLC Genomics Workbench (Version 7.0, Qiagen). The filtered raw reads from each BL sample were aligned to the WT-EBV (NC_007605) sequence using the CLC Genomics Workbench (version 7.0, Qiagen). Default parameters of mismatch cost of 2, insertion cost of 3, deletion cost of 3, length fraction of 1, and similarity fraction of 0.9 were used to obtain high-quality sequence alignment. The basic detection function was used to call nucleotide variations (single-nucleotide variations (SNVs) or multiple-nucleotide variations (MNVs), insertions and deletions in the reads with at least 5 reads at a particular base, and when the variant sequence appeared in at least 35% of the reads at that particular base. SNVs were categorized as synonymous or non-synonymous variations, depending on whether the variant coded for a different amino acid. Variation in the BL EBV genomes relative to WT EBV reference genome was quantified by dividing the number of variations in the particular genome by the total number of bases sequenced in that genome. Variations in the internal and terminal repeat regions of the EBV genome were disregarded.

Multiple sequence alignments of the 12 BL whole EBV genomes and 100 published EBV genomes (97 registered in the NCBI and 3 published from the 1000 Genomes Project)^38^, including 13 from tumor-derived BL cell lines, was done using the Kalign program (http://www.ebi.ac.uk/Tools/msa/kalign) installed on the NIH Helix supercomputer (https://helix.nih.gov) to facilitate phylogenetic analysis. An additional 18 EBV genomes marked as NPC-EBVs (AB850643 to AB850660) present in the NCBI database were not included in the whole genome analysis because they lacked complete references of publication, details about origin, or annotation. Individual gene alignments for LMP-1, EBNA-1 and BZLF1 proteins were analyzed by using the Clustal Omega program in EBI (http://www.ebi.ac.uk/Tools/msa/clustalo). The alignments were used to generate phylogenetic trees using Molecular Evolutionary Genetic Analysis (MEGA) software, version 5.0^47^ with a neighbor-joining algorithm.

### Data access

The full-length sequences of 12 BL EBV assembled genomes (supplemental Table 1): CCH, MP, SCL, VGO, RPF, CV-ARG, FNR, HU11393, H018436D, H058015C, H002213 and H03753A were annotated using the information derived from the reference genome WT-EBV (NC_007605.1) sequence. Results were submitted to the GenBank database with the accession numbers KP968257, KP968258, KP968259, KP968260, KR063344, KR063343, KR063345, KP968261, KP968262, KP968263 and KP968264, KR063342, respectively.

## Additional Information

**How to cite this article**: Lei, H. *et al.* Epstein-Barr virus from Burkitt Lymphoma biopsies from Africa and South America share novel LMP-1 promoter and gene variations. *Sci. Rep.* **5**, 16706; doi: 10.1038/srep16706 (2015).

## Supplementary Material

Supplementary Information

## Figures and Tables

**Table 1 t1:** Characteristics of the BL tumor samples that were sequenced for Epstein-Barr virus.

Sample ID (Subject ID)	GenBankaccessionnumber	Sex	Age,years	Tumor site	8;14abnormality
**Brazil**					
CCH	KP968257	M	4	Abdomen, CNS	Far 5’; NSμ
MP	KP968258	M	4	Abdomen	*H3-Pst*; Sμ
SCL	KP968259	F	6	Abdomen	*Sma-Pvu*- Sμ
VGO	KP968260	M	5	Abdomen	*H3-Pst*; Sμ
RPF	KR063344	M	11	Abdomen	Far 5’; NSμ
FNR	KR063345	M	6	Abdomen	Far 5’+; NSμ
**Argentina**					
CV-ARG	KR063343	F	7	Abdomen	Far 5’+; NSμJ_H_
**Ghana**					
HU11393 (K00091800)*	KP968261	M	7.23	Intestine	Not done
H03753A (K00091800)*	KR063342	M	7.23	Intestine	Not done
H018436D (K00062500)	KP968262	M	5.06	Colon	Not done
H058015C (K00126500)	KP968263	F	12.1	Abdomen	Not done
H002213 (K00094200)	KP968264	M	7.6 1	Spleen	Not done

*These samples were from the same subject, but different tumor sites.

**Table 2:**
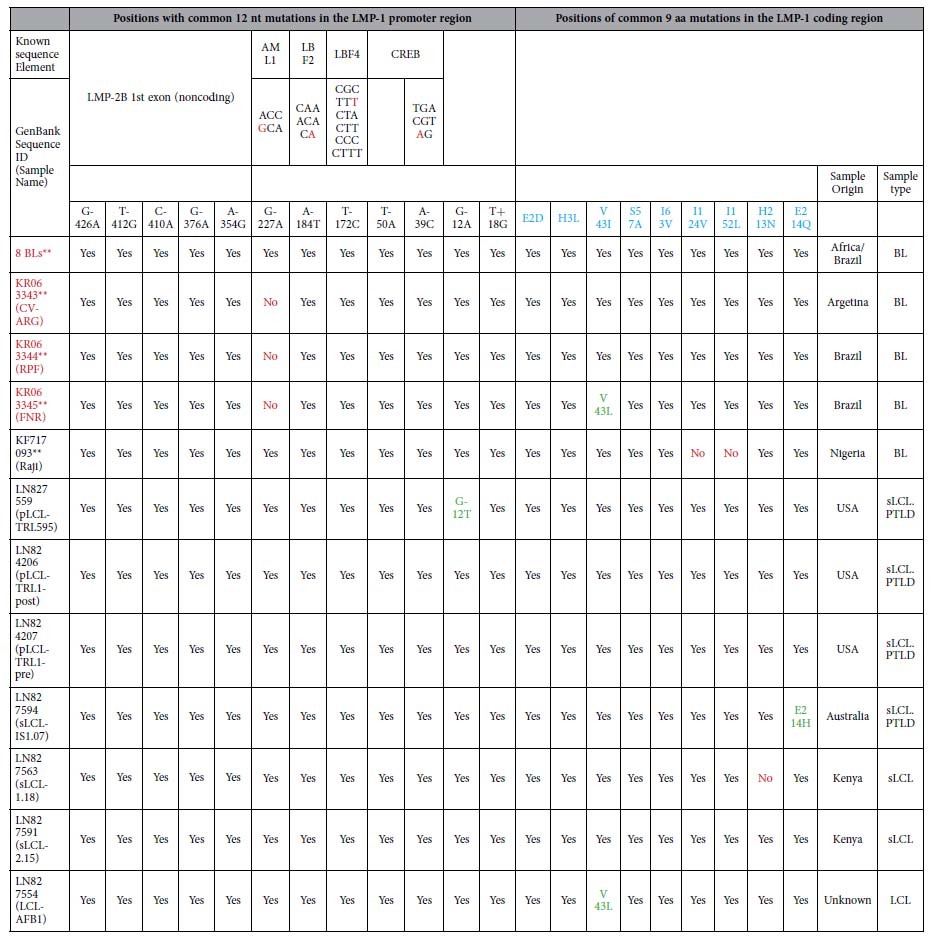
EBV genomes with the same or a highly similar pattern of nucleotide variations or mutations and imputed amino acid changes in the LMP-1 promoter and the coding regions.

Samples written in red font are EBV genomes sequenced directly form BL tumors in this study. **The eight BL cases are: 1) KP968258**(MP), 2) KP968259**(SCL), 3) KP968257**(CCH), 4) KP968262**(H018436D), 5) KP968263**(H058015C), 6) KP968261**(HU11393), 7) KR063342**(H03753A) and 8) KP968264**(H002213).
