# Supplementary material for: Epstein-Barr virus from Burkitt Lymphoma biopsies from Africa and South America share novel LMP-1 promoter and gene variations
Source: Sci Rep. 2015 Nov 23;5:16706. doi: 10.1038/srep16706 (PMC4655394; doi:10.1038/srep16706)
Supplement: Supplementary Information [file srep16706-s1.doc]

Supplementary Tables and Figures

Title: Epstein-Barr virus from Burkitt Lymphoma biopsies from Africa and South America share novel LMP-1 promoter and gene variations

Authors:

Haiyan Lei, Tianwei Li, Bingjie Li, Shien Tsai, Robert J Biggar, Francis Nkrumah, Janet Neequaye, Marina Gutierrez, Sidnei Epelman, Sam M Mbulaiteye, Kishor Bhatia^7^ and Shyh-Ching Lo

**Supplementary Table 1: Summary of sequence reads generated in genomic sequencing of BL tumors by Illumina MiSeq platform and reads mapped to WT-EBV genome**

| **Sample** | **No. of raw read** | **Through-put (Gb)** | **No. of EBV mapped reads** | **% of reads mapped to EBV** | **Average EBV genome coverage (folds)** | **% of reads mapped to human** | **Est. EBV copies per cell** | **No. of Gaps** | **Total Gap length (bp)** | **Assembled Genome length (bp)** |
| --- | --- | --- | --- | --- | --- | --- | --- | --- | --- | --- |
| **Brazil FNR** | **9,914,533** | **1.42** | **12,153** | **0.12** | **14.80** | **98.34** | **41.25** | **54** | **8,178** | **163,639** |
| **Brazil CCH** | **17,462,702** | **3.85** | **13,684** | **0.08** | **17.23** | **98.89** | **27.8** | **21** | **886** | **170,880** |
| **Brazil MP** | **20,856,806** | **4.43** | **57,812** | **0.28** | **69.66** | **98.57** | **95.4** | **4** | **116** | **171,595** |
| **Brazil SCL** | **17,828,596** | **3.75** | **21,166** | **0.12** | **24.37** | **98.21** | **39.76** | **24** | **2,227** | **169,587** |
| **Argentine CV-ARG** | **15,209,464** | **3.47** | **25,937** | **0.17** | **34.02** | **98.91** | **53.2** | **11** | **942** | **170,865** |
| **Brazil RPF** | **23,886,568** | **5.11** | **24,364** | **0.10** | **34.02** | **98.49** | **48.4** | **26** | **2,591** | **169,273** |
| **Brazil VGO** | **20,756,520** | **4.58** | **20,230** | **0.10** | **25.6** | **99.09** | **34.2** | **18** | **1,061** | **170,604** |
| **Ghana H018436D** | **16,823,300** | **3.52** | **31,167** | **0.19** | **38.37** | **98.87** | **66.1** | **5** | **217** | **171,577** |
| **Ghana H058015C** | **24,993,578** | **5.65** | **37,855** | **0.15** | **49.1** | **98.89** | **52.9** | **25** | **1,863** | **169,966** |
| **Ghana HU11393** | **16,780,482** | **3.83** | **43,976** | **0.26** | **58** | **98.62** | **92.2** | **6** | **476** | **171,329** |
| **Ghana H03753A** | **12,527,352** | **2.55** | **19,529** | **0.16** | **21.38** | **98.89** | **50.91** | **25** | **1,863** | **169,966** |
| **Ghana H002213** | **20,060,312** | **3.75** | **19,363** | **0.10** | **19.19** | **98.95** | **30.95** | **32** | **1,244** | **170,583** |

**Supplementary Table 2: Summary of variations identified in genomes of EBVs from 12 BL tumors in comparison with WT-EBV**

| **Sample** | **No. of variants** | **No. of SNVs** | **No. of MNVs** | **No. of insertions** | **No. of deletions** | **No. of coding region changes** | **No. of non-synonymous changes** |
| --- | --- | --- | --- | --- | --- | --- | --- |
| **Brazil FNR** | **397** | **373** | **7** | **8** | **9** | **363** | **120** |
| **Brazil CCH** | **517** | **489** | **14** | **5** | **9** | **493** | **162** |
| **Brazil MP** | **630** | **594** | **13** | **8** | **15** | **595** | **232** |
| **Brazil SCL** | **588** | **558** | **12** | **8** | **9** | **555** | **193** |
| **Argentine CV-ARG** | **696** | **648** | **21** | **7** | **20** | **662** | **226** |
| **Brazil RPF** | **876** | **800** | **29** | **21** | **23** | **845** | **287** |
| **Brazil VGO** | **1,160** | **1,073** | **38** | **21** | **23** | **1,115** | **361** |
| **Ghana H018436D** | **560** | **524** | **10** | **0** | **16** | **524** | **192** |
| **Ghana H058015C** | **546** | **522** | **10** | **6** | **8** | **514** | **176** |
| **Ghana HU11393** | **614** | **580** | **9** | **13** | **11** | **581** | **203** |
| **Ghana H03753A** | **522** | **494** | **9** | **11** | **8** | **499** | **164** |
| **Ghana H002213** | **531** | **505** | **10** | **9** | **7** | **513** | **180** |

**Supplementary Table 3: Analysis of EBV genome within each BL tumor for heterogeneity of nucleotide and amino acid sequence**

| **Sample** | **No. of site with sequence heterogeneity identified** | **Percentage in relation to the size of the genome** | **No. of site identified with high confidence** | **Position and amino acid change identified with high confidence** |
| --- | --- | --- | --- | --- |
| **Brazil FNR** | **10** | **0.008%** | **0** |  |
| **Brazil CCH** | **5** | **0.004%** | **0** |  |
| **Brazil MP** | **25** | **0.016%** | **1** | **140,419 of RPMS1, no aa change** |
| **Brazil SCL** | **8** | **0.005%** | **0** |  |
| **Argentine CV-ARG** | **22** | **0.015%** | **1** | **57,972 of BPLF1, no aa change** |
| **Brazil RPF** | **3** | **0.004%** | **0** |  |
| **Brazil VGO** | **32** | **0.019%** | **2** | **1) 5,048 of BNRF1, Ala1105Thr 2) 40,177 of LMP2, no aa change** |
| **Ghana HO18436D** | **15** | **0.01%** | **1** | **48,256 of BFRF2, Ala245Thr** |
| **Ghana HO58015C** | **17** | **0.01%** | **1** | **62,196 of BOLF1, no aa change** |
| **Ghana HU11393** | **7** | **0.005%** | **0** |  |
| **Ghana HO3753A** | **7** | **0.005%** | **0** |  |
| **Ghana HO02213** | **18** | **0.01%** | **0** |  |

**Supplementary Table 4: Detailed information of 112 EBV genomes registered with the NCBI included in phylogenetic analyses**, the source of tissue, country of origin, EBV Type and EBV pattern, based on the novel variations found in LMP-1 promoter (see text)

| **EBV Genome** | **Source** | **Origin** | **EBV-type** | **EBV Group using Novel LMP Patterns** |
| --- | --- | --- | --- | --- |
| KP968263**(H058015C) | BL biopsy | Ghana | Type1 | A |
| KP968262**(H018436D) | BL biopsy | Ghana | Type1 | A |
| KP968264**(H002213) | BL biopsy | Ghana | Type 1 | A |
| KP968261**(HU11393) | BL biopsy | Ghana | Type 1 | A |
| KR063342**(H03753A) | BL biopsy | Ghana | Type 1 | A |
| KR063345**(FNR) | BL biopsy | Brazil | Type 1 | A |
| KR063344**(RPF) | BL biopsy | Brazil | Type 1 | A |
| KP968258**(MP) | BL biopsy | Brazil | Type1 | A |
| KP968257**(CCH) | BL biopsy | Brazil | Type1 | A |
| KR063343**(CV-ARG) | BL biopsy | Arg | Type 1 | A |
| KP968259**(SCL) | BL biopsy | Brazil | Type 1 | A |
| LN827554(LCL-AFB1) | LCL | Unknown | Type 2 | A |
| LN824206(pLCL-TRL1-post) | sLCL. PTLD (pre) | USA | Type 1 | A |
| LN824207(pLCL-TRL1-pre) | sLCL. PTLD (post) | USA | Type 1 | A |
| LN827591(sLCL-2.15) | sLCL | Kenya | Type 2 | A |
| LN827594(sLCL-IS1.07) | sLCL. PTLD | Australia | Type 1 | A |
| LN827559(pLCL-TRL595) | sLCL. PTLD | USA | Type 1 | A |
| LN827563(sLCL-1.18) | sLCL | Kenya | Type 1 | A |
| KF717093**(Raji) | Raji | Nigeria | Type 1 | A |
|  |  |  |  |  |
| KP968260**(VGO) | BL biopsy | Brazil | Type 1 | B |
| KC207813**(Akata) | BL | Japan | Type1 | B |
| LN827525(C666-1) | NPC | Asia | Type1 | B |
| KJ411974(C666-1) | NPC | Asia | Type1 | B |
| KC617875(C666-1) | NPC | Asia | Type1 | B |
| AY961628(GD1) | NPC | China | Type1 | B |
| HQ020558(GD2) | NPC | China | Type1 | B |
| KF373730(M81) | NPC | Asia | Type1 | B |
| **LN824142(Saliva)** | Healthy saliva | UK | Type1 | B |
| **LN827562(sLCL-1.19)** | sLCL | Kenya | Type1 | B |
| **LN827561(YCCEL1)** | GC cell line | South Korea | Type1 | B |
| LN824209(HKN14) | sLCL | Hong Kong | Type1 | B |
| LN827547(HKN15) | sLCL | Hong Kong | Type1 | B |
| LN824224(HKN19) | sLCL | Hong Kong | Type1 | B |
| **LN827549(D3201.2)** | NPC | China | Type1 | B |
| JQ009376(HKNPC1) | NPC | Hong Kong | Type1 | B |
| KF992564(HKNPC2) | NPC | Hong Kong | Type1 | B |
| KF992565(HKNPC3) | NPC | Hong Kong | Type1 | B |
| KF992566(HKNPC4) | NPC | Hong Kong | Type1 | B |
| KF992567(HKNPC5) | NPC | Hong Kong | Type1 | B |
| KF992568(HKNPC6) | NPC | Hong Kong | Type1 | B |
| KF992569(HKNPC7) | NPC | Hong Kong | Type1 | B |
| KF992570(HKNPC8) | NPC | Hong Kong | Type1 | B |
| KF992571(HKNPC9) | NPC | Hong Kong | Type1 | B |
| LN827523(L591) | HL cell line | Germany | Type1 | B |
| **LN827799(sLCL-IM1.16)** | sLCL.IM | Australia | Type1 | B |
| LN827578(sLCL-IS1.13) | sLCL.PTLD | Australia | Type1 | B |
| **LN827586(sLCL-IS1.15)** | sLCL.PTLD | Australia | Type1 | B |
|  |  |  |  |  |
| LN827800**(Jijoye) | BL | Nigeria | Type2 | C |
| **LN827548**(P3HR1_c16)** | BL | Nigeria | Type2 | C |
| LN827557**(BL36) | BL | N. Africa | Type 1 | C |
| LN827545**(Daudi) | BL | Kenya | Type 1 | C |
| LN827551**(Makau) | BL | Kenya | Type 1 | C |
| LN824205(sLCL-1.12) | sLCL | Kenya | Type 1 | C |
| LN824203**(Mak1) | BL | Kenya | Type 1 | C |
|  |  |  |  |  |
| **LN827544**(Wewak1)** | BL | PNG | Type 2 | D |
| LN827556**(Cheptages) | BL | Kenya | Type 2 | D |
| **LN827526**(BL37)** | BL | Africa | Type1 | D |
| KC207814**(Mutu) | BL | Kenya | Type1 | D |
| NC_009334**(AG876) | BL | Ghana | Type2 | D |
| AJ507799(WT-EBV) | healthy donor | USA | Type 1 | D |
| V01555(B95-8) | healthy donor | USA | Type1 | D |
| KC440851(K4123-Mi) | healthy donor | USA | Type1 | D |
| KC440852(K4123-Mi) | healthy donor | USA | Type1 | D |
| NA19114 | healthy donor | Yoruba | Type 1 | D |
| NA19315 | healthy donor | Kenya | Type1 | D |
| NA19384 | healthy donor | Kenya | Type1 | D |
| LN827739(LCL_B958) | LCL, B95-8 | USA | Type1 | D |
| LN827597(sLCL-IS1.04) | sLCL. PTLD | Australia | Type1 | D |
| LN827596(sLCL-IM1.02) | sLCL. IM. | Australia | Type 1 | D |
| LN827595(sLCL-IS1.03) | sLCL. PTLD | Australia | Type1 | D |
| LN827593(sLCL-IS1.12) | sLCL. PTLD | Australia | Type1 | D |
| LN827592(sLCL-IS1.10) | sLCL. PTLD | Australia | Type1 | D |
| LN827590(sLCL-IM1.05) | sLCL. IM. | Australia | Type 1 | D |
| LN827589(sLCL-IS2.01) | sLCL. PTLD | Australia | Type2 | D |
| LN827588(sLCL-IS1.19) | sLCL. PTLD | Australia | Type1 | D |
| LN827587(sLCL-2.21) | sLCL | Kenya | Type 2 | D |
| LN827585(sLCL-1.04) | sLCL | Kenya | Type 1 | D |
| LN827584(sLCL-IS1.06) | sLCL. PTLD | Australia | Type1 | D |
| LN827583(sLCL-IM1.17) | sLCL. IM. | Australia | Type 1 | D |
| LN827582(sLCL-BL1.03) | sLCL | Kenya | Type 1 | D |
| LN827581(sLCL-1.05) | sLCL | Kenya | Type 1 | D |
| LN827580(sLCL-2.16) | sLCL | Kenya | Type 2 | D |
| LN827579(sLCL-1.13) | sLCL | Kenya | Type 1 | D |
| LN827577(sLCL-1.17) | sLCL | Kenya | Type 1 | D |
| LN827576(sLCL-IS1.20) | sLCL. PTLD | Australia | Type1 | D |
| LN827575(sLCL-IS1.14) | sLCL. PTLD | Australia | Type1 | D |
| LN827574(sLCL-1.09) | sLCL | Kenya | Type 1 | D |
| LN827573(sLCL-1.10) | sLCL | Kenya | Type 1 | D |
| LN827572(sLCL-IS1.18) | sLCL. PTLD | Australia | Type1 | D |
| LN827571(sLCL-BL1.20) | sLCL | Kenya | Type 1 | D |
| LN827570(sLCL-IS1.01) | sLCL. PTLD | Australia | Type1 | D |
| **LN827569(sLCL-IS1.11)** | sLCL. PTLD | Australia | Type1 | D |
| LN827568(sLCL-1.24) | sLCL | Kenya | Type 1 | D |
| LN827567(sLCL-IM1.09) | sLCL. IM. | Australia | Type 1 | D |
| LN827566(sLCL-1.06) | sLCL | Kenya | Type 1 | D |
| LN827565(sLCL-1.07) | sLCL | Kenya | Type 1 | D |
| **LN827564(HL04)** | HL | UK | Type 1 | D |
| LN827560(sLCL-2.14) | sLCL | Kenya | Type 2 | D |
| LN827558(sLCL-1.02) | sLCL | Kenya | Type 1 | D |
| LN827555(X50-7) | LCL | USA | Type 1 | D |
| LN827553(sLCL-IS1.08) | sLCL. PTLD | Australia | Type1 | D |
| LN827552(sLCL-1.08) | sLCL | Kenya | Type 1 | D |
| LN827550(sLCL-1.11) | sLCL | Kenya | Type 1 | D |
| **LN827546(HL02)** | HL | UK | Type 1 | D |
| LN827527(M-ABA) | LCL, NPC virus | N. Africa | Type 1 | D |
| LN827524(HL11) | HL | UK | Type 1 | D |
| **LN827522(HL09)** | HL | UK | Type 1 | D |
| **LN824226(HL01)** | HL | UK | Type 1 | D |
| **LN824225(HL08)** | HL | UK | Type 1 | D |
| **LN824204(HL05)** | HL | UK | Type 1 | D |


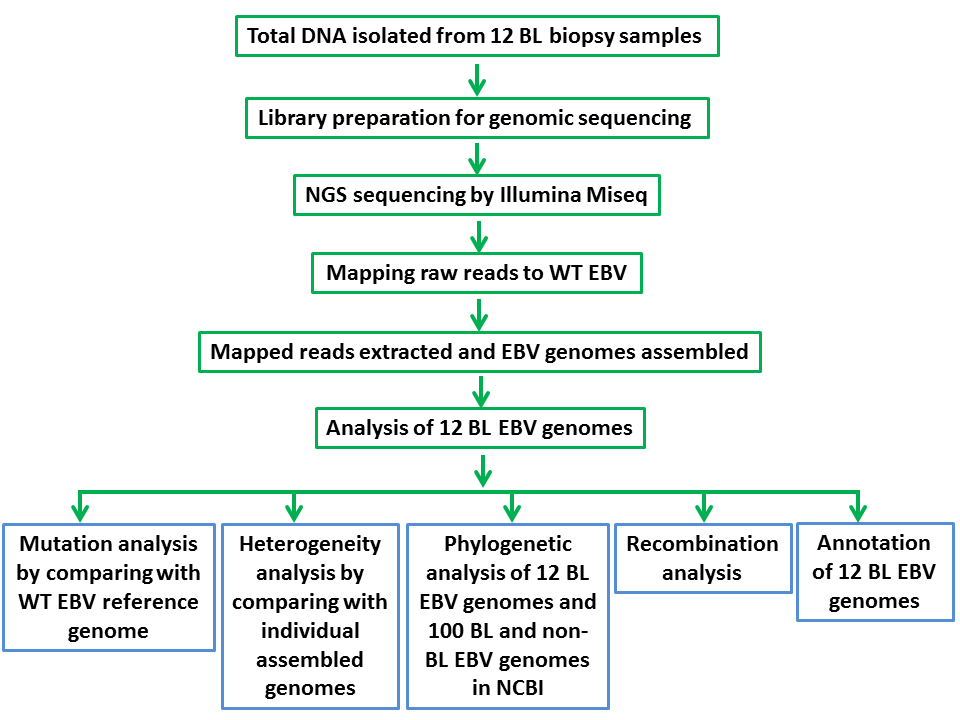
**Supplementary Figure 1:** Workflow for sequencing and analysis of 12 EBV genomes from Burkitt lymphoma biopsies

*Notes:* Input DNA extracted from primary BL tumors go through library preparation and NGS sequencing to generate sequence reads. Reads mapped to EBV were extracted and assembled to the EBV genome. Subsequent analysis was performed on the assembled EBV genomes.


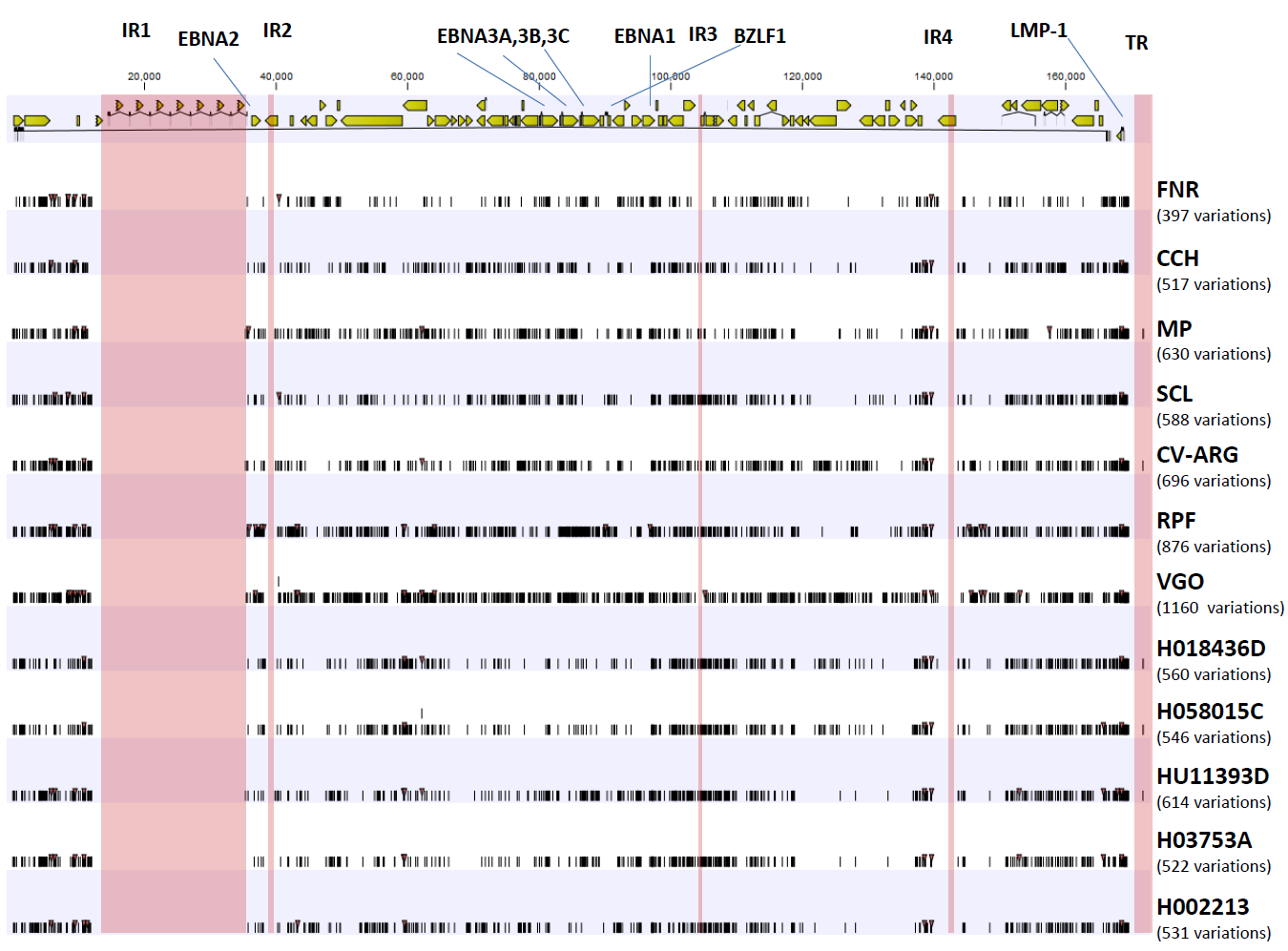
**Supplementary Figure 2: Nucleotide variations identified in the genomes of EBVs sequenced from BL tumors in comparison with WT-EBV (type 1) genome (A);with AG-876 genome of EBV (type-2) (B) and with with GD1-EBV genome (type 1) from NPC (C)**

**(A)**

**(B)**

**
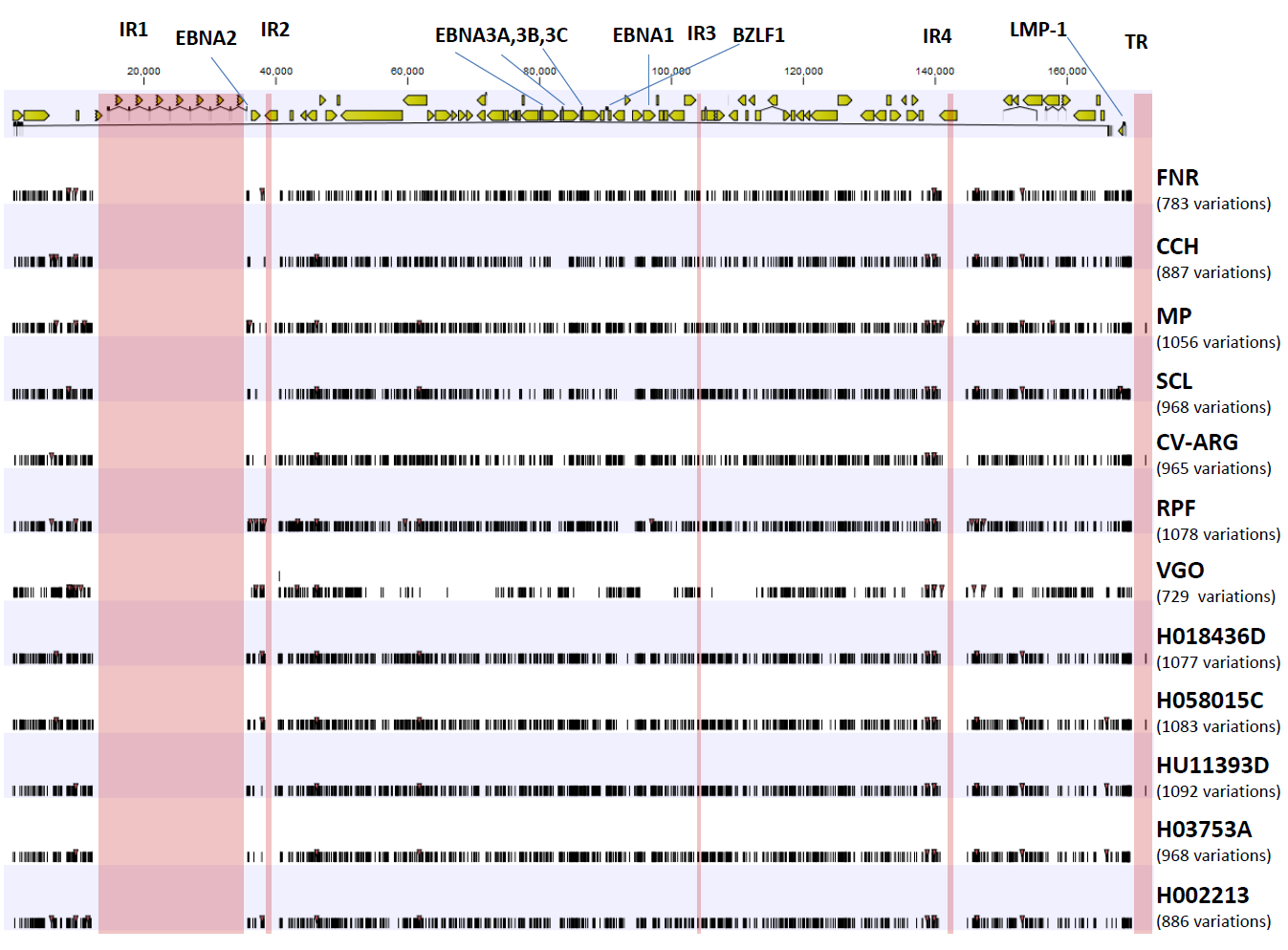
**

**(C )**

**
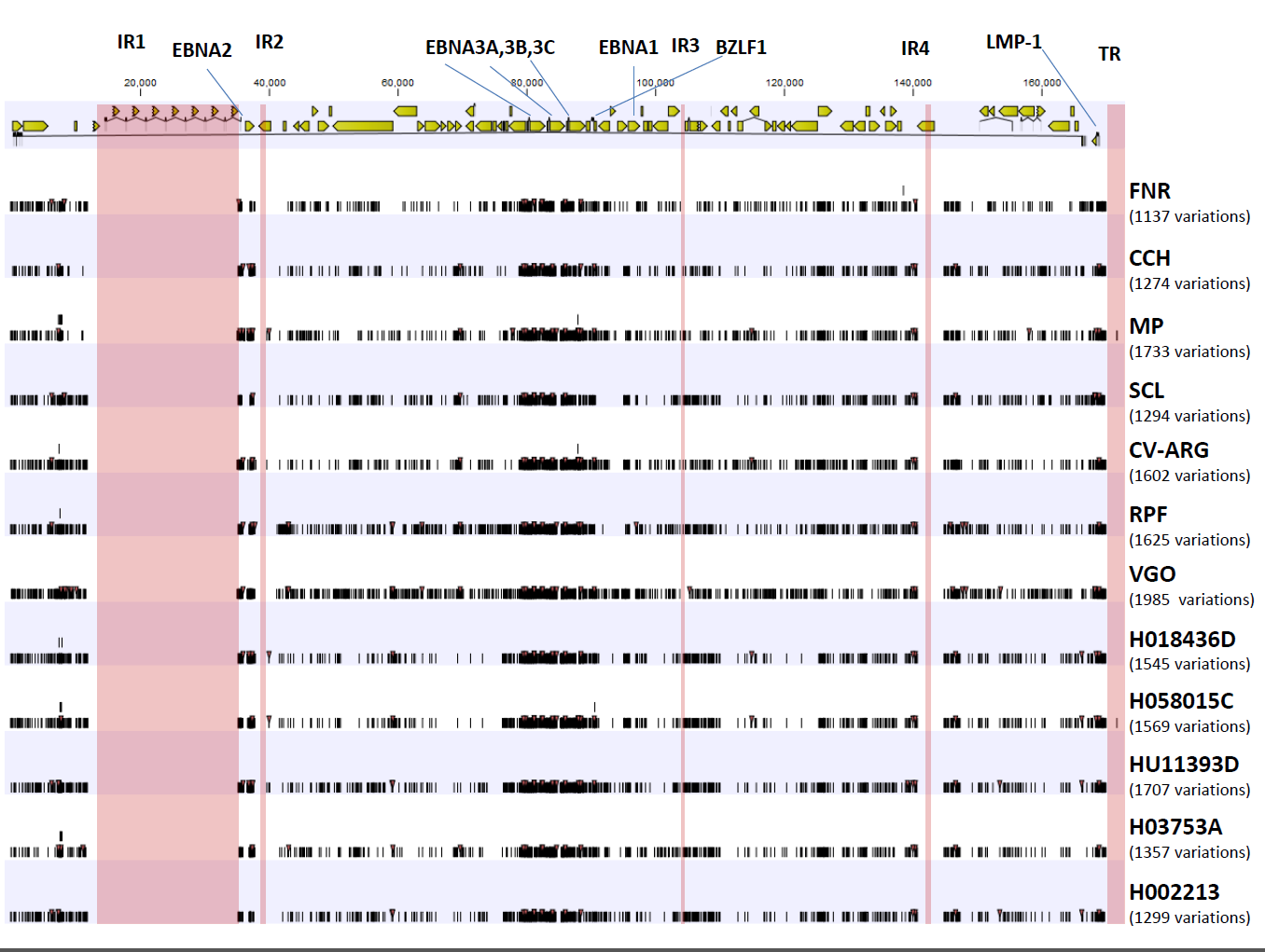
**
